# Time to Epidural Steroid Injection and Complete Remission in Zoster-Associated Pain: A Multicenter Retrospective Cohort Study

**DOI:** 10.3390/life16060869

**Published:** 2026-05-22

**Authors:** Yongsoo Lee, Eun Hee Chun, Hee Yong Kang, Harin Hong, Yeji Yang, Hye Sun Lee, Jung Eun Kim

**Affiliations:** 1 Department of Anesthesiology and Pain Medicine, Hallym University Kangnam Sacred Heart Hospital, Seoul 07441, Republic of Korea; thisisrio@naver.com (Y.L.);; 2 Department of Anesthesiology and Pain Medicine, Kyung Hee University College of Medicine, Kyung Hee University Hospital, Seoul 02447, Republic of Korea; 3 Biostatistics Collaboration Unit, Yonsei University College of Medicine, Seoul 03722, Republic of Korea

**Keywords:** allodynia, complete remission, epidural steroid injection, herpes zoster, hyperalgesia, neuralgia, postherpetic, time-to-treatment

## Abstract

Background: In zoster-associated pain (ZAP), earlier epidural steroid injection (ESI) has been associated with better outcomes, but optimal timing remains unclear, and prior studies have largely relied on pain reduction alone. Methods: In this multicenter retrospective cohort, 215 patients with ZAP who completed a three-session ESI course were classified into early (<30 days) and delayed (≥30–≤180 days) groups. The primary endpoint was complete remission at 12 weeks (≥50% visual analog scale [VAS] reduction, VAS ≤ 2, and sensory normalization); successful response (≥50% VAS reduction) served as the secondary endpoint. An ordered three-category framework and an exploratory generalized Youden index threshold analysis were applied. Results: Complete remission occurred in 82.1% versus 39.0% and successful response in 91.7% versus 67.8%. Each additional day of delay was associated with lower odds of complete remission (adjusted odds ratio [aOR], 0.957; *p* < 0.001) and higher odds of a worse outcome category (aOR, 1.030; *p* < 0.001). Exploratory candidate boundaries were 22 and 42 days. Conclusions: Earlier ESI initiation was associated with a higher likelihood of complete remission incorporating pain reduction, low residual pain intensity, and sensory normalization. These findings highlight the clinical relevance of treatment timing and recovery assessment beyond pain reduction alone in ZAP.

## 1. Introduction

Herpes zoster (HZ), caused by reactivation of latent varicella-zoster virus, imposes a substantial clinical burden largely through zoster-associated pain (ZAP), which may persist beyond the acute rash phase and impair sleep, daily functioning, and quality of life [1,2]. ZAP is commonly used as an umbrella term that includes acute herpetic pain in the early phase after rash onset, subacute pain, and postherpetic neuralgia (PHN), with PHN conventionally defined as pain persisting beyond 90 days after rash onset [3,4]. Because these phases may differ in the relative contributions of active inflammation, peripheral sensitization, and established neuropathic mechanisms, the timing of interventional treatment may be clinically relevant but remains uncertain. Although antiviral therapy accelerates rash healing and suppresses viral replication, its effect on preventing persistent neuropathic pain appears modest and inconsistent [5,6]. In patients with persistent or severe ZAP, additional pain-directed interventions may therefore be required, and epidural steroid injection (ESI) is commonly used [7,8].

However, pain reduction alone may not adequately capture clinically meaningful recovery in ZAP [9,10,11]. Even when pain intensity decreases, sensory abnormalities such as allodynia and hyperalgesia may persist, indicating incomplete recovery [12,13]. Accordingly, patients who meet conventional pain-based responder criteria may still differ meaningfully from those with low residual pain and normalized sensory findings [13,14,15]. These considerations highlight the need for outcome definitions that incorporate not only pain improvement but also low residual pain intensity and sensory normalization as markers of more complete recovery [9,10].

Against this background, two key clinical questions remain unresolved. First, although prior studies have commonly applied a conventional 30-day criterion and linked earlier ESI to better pain-related outcomes, it remains uncertain whether this criterion adequately captures clinically relevant timing patterns in recovery [16,17]. Second, because prior studies have largely relied on pain-intensity reduction alone to define treatment response, it remains uncertain whether a multidimensional endpoint incorporating low residual pain intensity and sensory normalization would better capture clinically meaningful recovery [9,11,13].

Accordingly, this study aimed to evaluate the association between time to ESI and 12-week recovery in patients with ZAP using a multidimensional recovery framework incorporating clinically meaningful pain reduction, low residual pain intensity, and sensory normalization. Complete remission, defined by all three criteria, was designated as the primary endpoint; successful response based on pain reduction alone served as a secondary endpoint for comparison with prior studies. Patients were also classified into an ordered three-category framework consisting of complete remission, partial response, and minimal response. Data-derived timing thresholds were examined as exploratory, hypothesis-generating analyses.

## 2. Materials and Methods

### 2.1. Study Design and Setting

This retrospective multicenter cohort study was conducted at the pain clinics of the Departments of Anesthesiology and Pain Medicine at two university-affiliated hospitals in Seoul, Republic of Korea: Hallym University Kangnam Sacred Heart Hospital and Kyung Hee University Hospital. Clinical data were extracted from the medical records of patients with ZAP who were treated between March 2018 and February 2025. The study was reported in accordance with the STROBE statement (Supplementary STROBE Checklist).

### 2.2. Participants

Consecutive patients with ZAP who underwent ESI at either participating institution during the study period were screened for eligibility. Patients were eligible if they met the following criteria: (1) age ≥ 18 years; (2) ZAP involving the cervical, thoracic, or lumbosacral dermatomes; (3) a documented date of HZ rash onset; (4) a baseline visual analog scale (VAS; 0 = no pain, 10 = worst pain imaginable) score ≥4 measured immediately before the first ESI [18]; and (5) initiation of the first ESI within 180 days after rash onset. Patients were excluded if they were immunocompromised or had undergone prior interventional pain procedures for the same ZAP episode.

For each eligible patient, the following variables were extracted from the medical records: age, sex, affected dermatome, baseline VAS score, date of HZ rash onset, date of the first ESI, time to ESI, and comorbidities. Time to ESI, defined as the interval from rash onset to the first ESI, served as the primary exposure variable. Rash onset was defined as the date of first appearance of vesicular rash documented in the medical record.

Inclusion in the final analytic cohort additionally required completion of the standardized three-session ESI course at baseline, week 2, and week 4 within the prespecified visit windows (±3 days for the second and third sessions) and availability of all prespecified outcome data. Required outcome data included VAS scores at baseline and at weeks 2, 4, 6, 8, and 12 (±3 days) and sensory assessments at baseline and week 12.

Information on concomitant ZAP-related medication use during follow-up was reviewed from the medical records. Gabapentinoids were prescribed in nearly all patients as part of routine care. However, additional analgesic and neuropathic pain medications were individualized and frequently modified during follow-up, including changes in dose, combinations, and duration. Because these medication exposures were heterogeneous, time-varying, and not protocolized, they were not prespecified for inclusion as stable covariates in the primary multivariable models.

### 2.3. Treatment Protocol

At both institutions, ESIs were performed by board-certified pain physicians using a harmonized fluoroscopy-guided interlaminar technique as part of a standardized three-session treatment course. As part of routine care, patients also received individualized pharmacologic management, including gabapentinoids and analgesics, and all had received standard antiviral therapy during the acute phase.

Each injectate consisted of dexamethasone (5 mg), 0.2% ropivacaine, and normal saline, to yield a final ropivacaine concentration of 0.1%. The total injectate volume was 6 mL for cervical procedures and 10 mL for thoracic or lumbosacral procedures. After sterile skin preparation with 2% chlorhexidine in alcohol and local infiltration with 1% lidocaine, an 18-gauge Tuohy needle was advanced into the epidural space using a saline loss-of-resistance technique. Epidural placement was confirmed by contrast spread using 0.5–1 mL of iohexol. Noninvasive blood pressure and pulse oximetry were monitored throughout the procedure, and patients were observed for at least 30 min afterward.

### 2.4. Outcome Measures

The primary outcome was complete remission at week 12, defined as ≥50% reduction in VAS score from baseline, VAS score ≤ 2, and absence of mechanical allodynia, cold allodynia, and hyperalgesia. This composite endpoint represented the most stringent level of recovery by integrating clinically meaningful pain reduction, low residual pain intensity, and sensory normalization [11,12,13,19,20]. The secondary outcome was successful response, defined as ≥50% reduction in VAS score from baseline at week 12 regardless of sensory status [11]. Longitudinal pain intensity was assessed using VAS scores at baseline and at weeks 2, 4, 6, 8, and 12.

Patients were further classified into three mutually exclusive ordered categories: complete remission, partial response (successful response without complete remission), and minimal response (<50% reduction in VAS score from baseline at week 12). Outcome assessments were harmonized across both institutions. Sensory signs were assessed at baseline and week 12 by treating pain physicians using a standardized bedside protocol: mechanical allodynia with a filament brush, cold allodynia with an alcohol-soaked cotton swab, and mechanical hyperalgesia by pinprick with a 26-gauge needle [13,15,21]. De-identified sensory descriptors were independently adjudicated by two attending pain physicians according to predefined criteria; inter-rater agreement before consensus resolution was high (Cohen’s κ = 0.87), and disagreements were resolved by a third adjudicator.

### 2.5. Statistical Analysis

The primary and secondary endpoints were predefined; the threshold analysis was considered exploratory and hypothesis-generating. Data from both institutions were pooled because clinical and assessment protocols were harmonized. To examine the clinical relevance of the conventional 30-day criterion, patients were categorized into an early ESI group (<30 days) and a delayed ESI group (≥30 to ≤180 days) [13,16,22]. Normality of continuous variables was assessed using the Shapiro–Wilk test. Continuous variables were summarized as median (interquartile range [IQR]) and compared using the Mann–Whitney U test; categorical variables were presented as number (%) and compared using the chi-square or Fisher’s exact test.

Longitudinal VAS trajectories were analyzed using a linear mixed model (LMM) with an unstructured covariance matrix. Fixed effects included group, time, group × time interaction, age, and sex. Estimated marginal means (EMMs) were derived at each time point. Planned contrasts, including interaction contrasts, were used to compare groups and assess changes across intervals, with Bonferroni adjustment for post hoc comparisons.

To explore data-derived timing patterns beyond the conventional 30-day criterion, a three-category threshold analysis was performed using the generalized Youden index [23]. This analysis was considered exploratory and hypothesis-generating. Dual thresholds were identified for the ordered three-category outcome. This threshold analysis was performed without adjustment for age and sex.

To quantify the association between treatment timing and 12-week outcomes across the full timing range, binary logistic regression was used for complete remission and successful response, and ordinal logistic regression was used for the ordered three-category outcome, with time to ESI treated as a continuous variable. All models were adjusted for age and sex. Baseline VAS score was not included because both binary and ordered outcomes incorporated change from baseline through the requirement for ≥50% VAS reduction. The proportional odds assumption was assessed using the score test.

Baseline characteristics across the three ordered outcome categories were compared using the Kruskal–Wallis test for continuous variables and the chi-square or Fisher’s exact test for categorical variables. Post hoc pairwise comparisons used Dunn’s test with Bonferroni correction.

All analyses were conducted on a complete-case basis using SAS version 9.4 (SAS Institute Inc., Cary, NC, USA) and R version 4.3.2 (R Foundation for Statistical Computing, Vienna, Austria). No imputation was performed for missing data. All tests were two-sided, and *p* < 0.05 was considered statistically significant.

## 3. Results

### 3.1. Study Population

**Figure 1:**
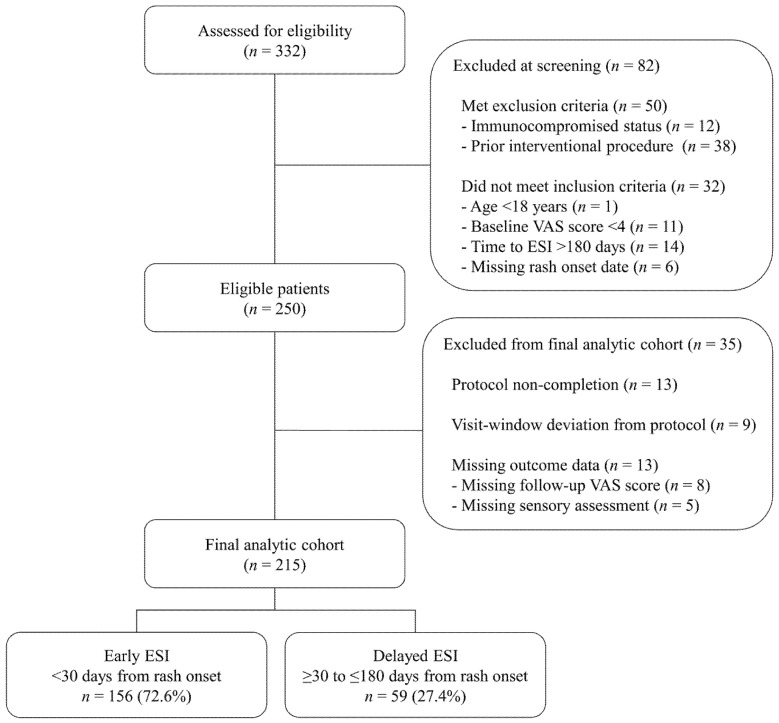
Flow of patient selection. Of 332 consecutive patients with ZAP assessed during the study period, 82 were excluded at screening, leaving 250 eligible patients. Of these, 35 were excluded from the final analytic cohort because of protocol non-completion, visit-window deviations, or missing prespecified outcome data, leaving 215 patients for analysis. The final analytic cohort was classified into the early ESI group (<30 days from rash onset; *n* = 156) and the delayed ESI group (≥30 to ≤180 days from rash onset; *n* = 59). ESI, epidural steroid injection; VAS, visual analog scale; ZAP, zoster-associated pain.

Of 332 consecutive patients with ZAP screened during the study period, 82 did not meet the eligibility criteria. An additional 35 eligible patients were excluded from the final analytic cohort because of protocol non-completion, visit-window deviations from protocol, or missing prespecified outcome data, leaving 215 patients for analysis (Figure 1). Patients were classified into an early ESI group (<30 days; *n* = 156, 72.6%) and a delayed ESI group (≥30 to ≤180 days; *n* = 59, 27.4%) based on the conventional 30-day criterion. No procedure-related complications requiring further intervention were observed.

### 3.2. Baseline Characteristics

Baseline characteristics are presented in Table 1. Patients in the early ESI group were significantly younger than those in the delayed ESI group (median 63.0 years [IQR, 53.5–72.0] vs. 71.0 years [IQR, 62.0–77.0]; *p* = 0.001). The proportion of male patients did not differ significantly between groups (43.6% vs. 45.8%; *p* = 0.775). Baseline pain intensity was comparable between groups (median VAS score, 8.0 [IQR, 6.0–8.0] vs. 8.0 [IQR, 6.0–9.0]; *p* = 0.378). The thoracic dermatome was the most commonly affected level in both groups (61.5% vs. 74.6%), and dermatomal distribution did not differ significantly (*p* = 0.140). No significant differences were observed in the assessed comorbidities between groups.

### 3.3. Twelve-Week Outcomes According to the Conventional 30-Day Criterion

At 12 weeks, complete remission was achieved in 151 of 215 patients (70.2%) overall and was significantly more frequent in the early ESI group than in the delayed ESI group (82.1% vs. 39.0%; risk difference [RD], 43.1 percentage points [95% CI, 28.6–55.6]; risk ratio [RR], 2.10 [95% CI, 1.52–2.92]; *p* < 0.001). Successful response was observed in 183 of 215 patients (85.1%) and was significantly more frequent in the early ESI group (91.7% vs. 67.8%; RD, 23.9 percentage points [95% CI, 12.0–37.0]; RR, 1.35 [95% CI, 1.13–1.62]; *p* < 0.001). The absolute between-group difference was more pronounced for complete remission than for successful response.

### 3.4. Longitudinal VAS Trajectories According to the Conventional 30-Day Criterion

**Figure 2:**
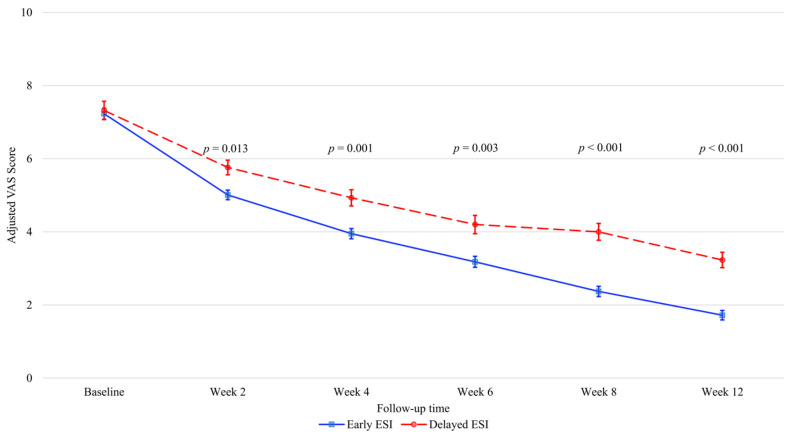
Adjusted longitudinal VAS trajectories according to the conventional 30-day criterion. Adjusted VAS scores are presented as EMMs ± SEs derived from the age- and sex-adjusted LMM. Patients were classified into the early ESI group (<30 days from rash onset) and the delayed ESI group (≥30 to ≤180 days from the rash onset). Adjusted baseline VAS scores did not differ between groups, whereas the early ESI group showed significantly lower adjusted VAS scores at each follow-up assessment. The *p*-values shown in the figure indicate between-group comparisons at each follow-up time point. EMM, estimated marginal means; ESI, epidural steroid injection; LMM, linear mixed model; SE, standard error; VAS, visual analog scale.

Longitudinal VAS trajectories according to the conventional 30-day criterion are shown in Figure 2. In the age- and sex-adjusted linear mixed model, group, time, and the group × time interaction were all significant (all *p* < 0.001), indicating different longitudinal recovery trajectories between the early and delayed ESI groups. Age was significantly associated with longitudinal VAS scores (*p* < 0.001), whereas sex was not (*p* = 0.933). Adjusted baseline EMMs did not differ between groups (7.23 vs. 7.32; *p* > 0.999), consistent with the comparable baseline pain intensity shown in Table 1. However, the early ESI group had significantly lower adjusted VAS scores at all follow-up time points, including week 2 (5.01 vs. 5.76; *p* = 0.013), week 4 (3.95 vs. 4.93; *p* = 0.001), week 6 (3.18 vs. 4.20; *p* = 0.003), week 8 (2.37 vs. 4.00; *p* < 0.001), and week 12 (1.72 vs. 3.23; *p* < 0.001).

In the early ESI group, VAS scores decreased significantly across all consecutive intervals. By contrast, in the delayed ESI group, the change during the week 6–8 interval was not significant (*p* > 0.999). Interaction contrasts confirmed greater cumulative pain reduction in the early group from baseline to weeks 2, 4, 6, 8, and 12 (all *p* ≤ 0.047), and from week 2 to weeks 8 and 12 (*p* = 0.006 and *p* = 0.034, respectively).

### 3.5. Three-Category Threshold Analysis

**Figure 3:**
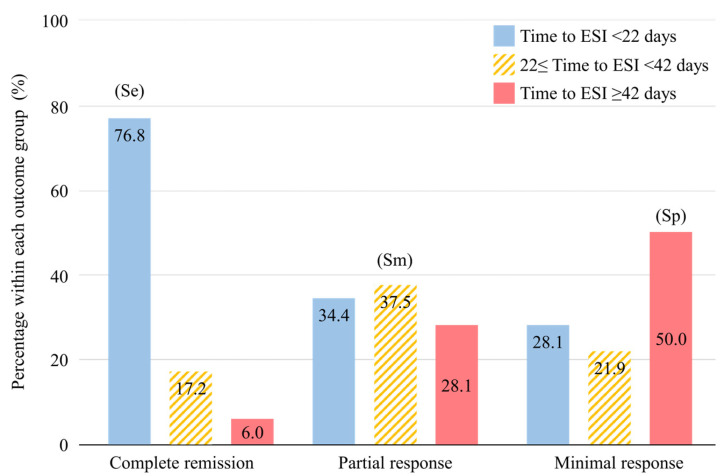
Distribution of the data-derived time to ESI groups across the ordered three-category outcome framework. Bars show the percentage distribution of the three exploratory time to ESI groups within each outcome category: <22 days, ≥22 to <42 days, and ≥42 days. Se denotes the correct classification rate for complete remission, defined as the proportion of patients with complete remission whose time to ESI was <22 days. Sm denotes the correct classification rate for partial response, defined as the proportion of patients with partial response whose time to ESI was ≥22 to <42 days. Sp denotes the correct classification rate for minimal response, defined as the proportion of patients with minimal response whose time to ESI was ≥42 days. ESI, epidural steroid injection.

Exploratory threshold analysis was performed to identify data-derived timing boundaries beyond the conventional 30-day criterion (Figure 3). In the three-category threshold analysis using the generalized Youden index, dual thresholds of 22 and 42 days defined three timing groups: <22 days (*n* = 136), ≥22 to <42 days (*n* = 45), and ≥42 days (*n* = 34). Among patients with complete remission, most had time to ESI < 22 days; among those with partial response, the highest proportion had time to ESI ≥ 22 to <42 days; and among those with minimal response, the highest proportion had time to ESI ≥ 42 days.

### 3.6. Continuous and Three-Category Associations of Time to ESI with 12-Week Outcomes

To further quantify the relationship between treatment timing and 12-week outcomes, Table 2 presents the associations of time to ESI, modeled as a continuous variable, with the binary and ordered outcomes. In unadjusted analyses, time to ESI was the only examined variable significantly associated with all analyzed outcomes, whereas no other examined variables showed a significant association with these outcomes (Supplementary Table S1).

For complete remission, each additional day of delay was associated with lower odds of complete remission in unadjusted analysis (OR 0.957; 95% CI, 0.942–0.972; *p* < 0.001), and this association remained after adjustment for age and sex (adjusted OR [aOR] 0.957; 95% CI, 0.941–0.972; *p* < 0.001). For successful response, each additional day of delay was also associated with lower odds of response in both unadjusted (OR 0.976; 95% CI, 0.965–0.988; *p* < 0.001) and adjusted analyses (aOR 0.978; 95% CI, 0.966–0.990; *p* < 0.001).

In ordinal logistic regression for the three-category outcome, in which higher tiers indicate worse recovery, each additional day of delay was associated with higher odds of being in a worse outcome tier in both unadjusted analysis (OR 1.030; 95% CI, 1.019–1.042; *p* < 0.001) and the adjusted model (aOR 1.030; 95% CI, 1.018–1.041; *p* < 0.001). Age and sex were not significantly associated with the ordinal outcome, and the proportional odds assumption was not violated (score test, *p* = 0.796).

Consistent with these regression findings, comparison across the three 12-week outcome groups showed that only time to ESI differed significantly (Table 3; *p* < 0.001). The shortest interval was observed in the complete remission group (*n* = 151; median 10.0 days [IQR, 6.0–21.0]), followed by the partial response group (*n* = 32; median 30.0 days [IQR, 7.0–62.0]) and the minimal response group (*n* = 32; median 39.0 days [IQR, 15.0–91.0]). Dunn-adjusted pairwise comparisons showed that the complete remission group differed significantly from both the partial response group (*p* = 0.002) and the minimal response group (*p* < 0.001), whereas the partial response and minimal response groups did not differ significantly (*p* = 0.635). No significant differences were observed across the three groups for age, sex, dermatomal level, or assessed comorbidities.

## 4. Discussion

In this multicenter retrospective cohort study, earlier ESI was significantly associated with more favorable 12-week outcomes in patients with ZAP. Importantly, this timing-outcome association was more pronounced when recovery was defined as complete remission—incorporating clinically meaningful pain reduction, low residual pain intensity, and sensory normalization—than when defined by pain reduction alone. This pattern highlights the value of incorporating sensory normalization alongside pain reduction in defining recovery in ZAP.

These findings are broadly consistent with previous studies showing that earlier interventional treatment is associated with better outcomes in ZAP. Kim et al. reported that patients who underwent ESI within 30 days after herpes zoster onset achieved complete pain relief faster than those treated between 30 and 90 days [16]. Similarly, Nahm et al. identified symptom duration of ≤12 weeks as the only significant predictor of favorable outcome after ESI [17]. In addition, related interventional studies have shown that early spinal nerve root block administered within 14 days of HZ onset reduces both the incidence and duration of PHN [24]. Taken together, these studies suggest that treatment timing may be clinically relevant, but the clinically most informative treatment window within the early phase has not been clearly defined, and outcome assessment has largely relied on pain intensity alone [13,16,17].

Allodynia and hyperalgesia are clinically important sensory manifestations of zoster-associated neuropathic pain [12,13,15]. Their persistence despite improvement in pain intensity may indicate incomplete clinical recovery [12,13,15], supporting the inclusion of sensory normalization in recovery assessment [9,10,11,13]. In the present study, ESI initiation within 30 days was associated with a higher likelihood of complete remission at 12 weeks. The between-group difference was larger for complete remission than for successful response based on pain reduction alone. These findings suggest that a composite endpoint incorporating clinically meaningful pain reduction, low residual pain intensity, and sensory normalization provides a more stringent and clinically informative assessment of recovery.

The longitudinal VAS analysis provided complementary evidence for the timing–outcome association. The early ESI group had consistently lower adjusted VAS scores at all follow-up time points and demonstrated significant improvement across consecutive intervals, whereas the delayed group showed no significant improvement between weeks 6 and 8. These longitudinal patterns, together with the higher rate of complete remission, support the clinical relevance of treatment timing in ZAP and the value of incorporating sensory normalization in recovery assessment. However, these findings reflect observational associations and should not be interpreted as evidence of a direct causal effect of earlier ESI.

Extending these observations, the exploratory threshold analysis suggested possible timing patterns beyond the conventional 30-day criterion. Using a generalized Youden index-based three-category approach, 22 and 42 days were identified as candidate boundaries for the ordered recovery categories. These data-derived boundaries corresponded to the observed distribution of complete remission, partial response, and minimal response, but they were derived from a single cohort without internal validation. Therefore, they should be regarded as hypothesis-generating and require confirmation in independent cohorts before clinical application.

Within this framework, the ordered three-category analysis supported a graded association between time to ESI and recovery status. Longer time to ESI was associated with higher odds of a worse recovery category, and among the examined variables, time to ESI was the only variable that differed significantly across the three outcome groups. These findings suggest that an ordered recovery framework may provide complementary information beyond a binary response definition based on pain reduction alone. However, these findings should be interpreted in light of potential residual confounding. Patients who received earlier ESI may have differed systematically from those treated later in ways not captured by the available covariates, including symptom severity at presentation, healthcare access, referral pathways, physician or patient preferences, and other unmeasured clinical factors. Differences in the natural course of ZAP may also have influenced both referral timing and eventual recovery. Thus, the observed associations should not be interpreted as evidence that earlier ESI directly caused better recovery.

This study has several strengths. First, it used a stringent recovery framework that incorporated low residual pain intensity and sensory normalization in addition to pain reduction, allowing recovery in ZAP to be assessed beyond conventional responder criteria. Second, it examined the conventional 30-day criterion together with exploratory data-derived timing thresholds, thereby generating hypotheses about possible timing patterns in recovery. Third, the multicenter design was supported by harmonized protocols across two university-affiliated institutions, including a standardized three-session ESI regimen and standardized sensory outcome assessment with high inter-rater agreement. In addition, the use of longitudinal trajectory analysis, regression analyses treating time to ESI as a continuous variable, ordinal regression, and exploratory threshold-based analysis provided complementary perspectives on the association between treatment timing and recovery.

Several limitations should be acknowledged. First, the retrospective observational design precludes causal inference. Treatment timing was not randomized, and residual confounding and indication bias cannot be excluded despite adjustment for age and sex. Second, the analytic cohort was restricted to patients who completed the three-session ESI protocol and had all prespecified outcome data available, which may have introduced selection bias. Third, concomitant pharmacologic management was individualized rather than protocolized; gabapentinoids were prescribed in nearly all patients, and the heterogeneity and time-varying nature of additional agents precluded their reliable inclusion as covariates in the primary models. Fourth, the exploratory 22- and 42-day thresholds were derived without internal validation procedures such as bootstrapping or split-sample validation and may therefore be sample-dependent; external validation in independent cohorts is required before clinical application. Finally, the 12-week follow-up does not allow assessment of longer-term outcomes; prospective studies with extended follow-up will be needed to determine whether the observed differences in complete remission and sensory normalization persist.

## 5. Conclusions

Earlier initiation of ESI was associated with a higher likelihood of complete remission, defined by clinically meaningful pain reduction, low residual pain intensity, and sensory normalization. These findings support the clinical relevance of treatment timing in ZAP recovery and highlight the value of assessing recovery beyond pain reduction alone. Exploratory timing findings from this study may help inform future prospective research aimed at refining clinically applicable treatment windows.

## Figures and Tables

**Table 1:** Baseline characteristics according to the conventional 30-day criterion.

Variables	Overall (*n =* 215)	Early ESI (*n =* 156)	Delayed ESI (*n =* 59)	*p*-Value
Demographics				
Age, years	65.0 (57.0–73.0)	63.0 (53.5–72.0)	71.0 (62.0–77.0)	0.001
Sex, male	95 (44.2)	68 (43.6)	27 (45.8)	0.775
Baseline VAS score	8.0 (6.0–8.0)	8.0 (6.0–8.0)	8.0 (6.0–9.0)	0.378
Dermatomal level				0.140
Cervical	47 (21.9)	36 (23.1)	11 (18.6)	
Thoracic	140 (65.1)	96 (61.5)	44 (74.6)	
Lumbosacral	28 (13.0)	24 (15.4)	4 (6.8)	
Comorbidities				
Hypertension	75 (34.9)	60 (38.5)	15 (25.4)	0.074
Diabetes mellitus	33 (15.3)	24 (15.4)	9 (15.3)	0.981
Heart disease	19 (8.8)	16 (10.3)	3 (5.1)	0.233
Thyroid disease	11 (5.1)	10 (6.4)	1 (1.7)	0.296
CVA	7 (3.3)	6 (3.9)	1 (1.7)	0.677
Asthma	4 (1.9)	3 (1.9)	1 (1.7)	>0.999
Hyperlipidemia	3 (1.4)	2 (1.3)	1 (1.7)	>0.999

Values are presented as median (IQR) or *n* (%). Continuous variables were compared using the Mann–Whitney U test, and categorical variables were compared using the chi-square test or Fisher’s exact test, as appropriate. Patients were classified according to the conventional 30-day criterion into the early ESI group (<30 days from rash onset) and delayed ESI group (≥30 to ≤180 days from rash onset). CVA, cerebrovascular accident; ESI, epidural steroid injection; IQR, interquartile range; VAS, visual analog scale.

**Table 2:** Continuous associations of time to ESI with 12-week treatment outcomes.

Variable	Unadjusted OR (95% CI)	*p*-Value	Adjusted OR (95% CI)	*p*-Value
A. Complete remission				
Time to ESI (per day)	0.957 (0.942–0.972)	<0.001	0.957 (0.941–0.972)	<0.001
Age (per year)	0.984 (0.963–1.006)	0.153	1.002 (0.977–1.027)	0.894
Sex (male vs. female)	1.025 (0.569–1.848)	0.933	1.007 (0.515–1.968)	0.983
B. Successful response				
Time to ESI (per day)	0.976 (0.965–0.988)	<0.001	0.978 (0.966–0.990)	<0.001
Age (per year)	0.985 (0.958–1.014)	0.306	0.993 (0.961–1.025)	0.663
Sex (male vs. female)	1.187 (0.553–2.546)	0.660	1.185 (0.520–2.700)	0.686
C. Three-category outcome (ordinal logistic regression)				
Time to ESI (per day)	1.030 (1.019–1.042)	<0.001	1.030 (1.018–1.041)	<0.001
Age (per year)	1.016 (0.994–1.038)	0.147	1.004 (0.981–1.028)	0.735
Sex (male vs. female)	0.951 (0.532–1.699)	0.865	0.993 (0.536–1.841)	0.983

Values are presented as unadjusted ORs or adjusted ORs with 95% CIs. Panels A and B were analyzed using binary logistic regression, and Panel C was analyzed using ordinal logistic regression. Age and sex were included in all adjusted models as predefined covariates. Complete remission was defined as ≥50% reduction in VAS score from baseline, VAS ≤ 2, and absence of mechanical allodynia, cold allodynia, and hyperalgesia at 12 weeks. Successful response was defined as ≥50% reduction in VAS score from baseline at 12 weeks regardless of sensory status. In Panel C, OR > 1 indicates higher odds of belonging to a worse recovery category for each additional day of delay in time to ESI. CI, confidence interval; ESI, epidural steroid injection; OR, odds ratio; VAS, visual analog scale.

**Table 3:** Characteristics according to the three-category outcome framework.

Variables	Complete Remission (*n =* 151)	Partial Response (*n =* 32)	Minimal Response (*n =* 32)	*p*-Value
Age, years	64.0 (55.0–73.0)	64.0 (59.0–74.0)	69.5 (60.5–74.0)	0.380
Time to ESI, days	10.0 (6.0–21.0)	30.0 (7.0–62.0)	39.0 (15.0–91.0)	<0.001
Sex, male	67 (44.4)	15 (46.9)	13 (40.6)	0.878
Dermatomal level				0.868
Cervical	32 (21.2)	6 (18.8)	9 (28.1)	
Thoracic	100 (66.2)	21 (65.6)	19 (59.4)	
Lumbosacral	19 (12.6)	5 (15.6)	4 (12.5)	
Comorbidities				
Hypertension	49 (32.5)	12 (37.5)	14 (43.8)	0.450
Diabetes mellitus	20 (13.2)	4 (12.5)	9 (28.1)	0.125
Heart disease	14 (9.3)	2 (6.3)	3 (9.4)	0.934
Thyroid disease	9 (6.0)	1 (3.1)	1 (3.1)	>0.999
CVA	4 (2.6)	1 (3.1)	2 (6.3)	0.586
Asthma	3 (2.0)	1 (3.1)	0 (0.0)	0.760
Hyperlipidemia	1 (0.7)	2 (6.3)	0 (0.0)	0.117

Values are presented as median (IQR) or *n* (%). Continuous variables were compared using the Kruskal–Wallis test, and categorical variables were compared using the chi-square test or Fisher’s exact test, as appropriate. The ordered three-category outcome framework consisted of complete remission, partial response, and minimal response. Complete remission was defined as ≥50% reduction in VAS score from baseline, VAS score ≤ 2, and absence of mechanical allodynia, cold allodynia, and hyperalgesia at 12 weeks. Partial response was defined as successful response without complete remission, and minimal response was defined as <50% reduction in VAS score from baseline at 12 weeks. For time to ESI, Dunn-adjusted pairwise comparisons were significant for complete remission versus partial response (*p* = 0.002) and complete remission versus minimal response (*p* < 0.001), but not for partial response versus minimal response (*p* = 0.635). CVA, cerebrovascular accident; ESI, epidural steroid injection; IQR, interquartile range; VAS, visual analog scale.

## Data Availability

The data that support the findings of this study are available from the corresponding author upon reasonable request.

## Supplementary Materials

The following supporting information can be downloaded at: https://www.mdpi.com/article/10.3390/life16060869/s1, Supplementary STROBE Checklist; Table S1: Unadjusted binary and ordinal logistic regression results for additional candidate variables.

## Abbreviations

The following abbreviations are used in this manuscript:

aOR	Adjusted odds ratio
CI	Confidence interval
CVA	Cerebrovascular accident
EMM	Estimated marginal mean
ESI	Epidural steroid injection
HZ	Herpes zoster
IQR	Interquartile range
LMM	Linear mixed model
OR	Odds ratio
PHN	Postherpetic neuralgia
RD	Risk difference
RR	Risk ratio
VAS	Visual analog scale
ZAP	Zoster-associated pain

## References

[1] Kawai K., Gebremeskel B.G., Acosta C.J. (2014). Systematic review of incidence and complications of herpes zoster: Towards a global perspective. BMJ Open.

[2] Drolet M., Brisson M., Schmader K.E., Levin M.J., Johnson R., Oxman M.N., Patrick D., Blanchette C., Mansi J.A. (2010). The impact of herpes zoster and postherpetic neuralgia on health-related quality of life: A prospective study. CMAJ.

[3] Dworkin R.H., Portenoy R.K. (1996). Pain and its persistence in herpes zoster. Pain.

[4] Johnson R.W., Rice A.S.C. (2014). Postherpetic neuralgia. N. Engl. J. Med..

[5] Chen N., Li Q., Yang J., Zhou M., Zhou D., He L. (2014). Antiviral treatment for preventing postherpetic neuralgia. Cochrane Database Syst. Rev..

[6] Wood M.J., Kay R., Dworkin R.H., Soong S.-J., Whitley R.J. (1996). Oral acyclovir therapy accelerates pain resolution in patients with herpes zoster: A meta-analysis of placebo-controlled trials. Clin. Infect. Dis..

[7] Kim H.J., Ahn H.S., Lee J.Y., Choi S.S., Cheong Y.S., Kwon K., Yoon S.H., Leem J.G. (2017). Effects of applying nerve blocks to prevent postherpetic neuralgia in patients with acute herpes zoster: A systematic review and meta-analysis. Korean J. Pain.

[8] van Wijck A.J.M., Opstelten W., Moons K.G.M., van Essen G.A., Stolker R.J., Kalkman C.J., Verheij T.J.M. (2006). The PINE study of epidural steroids and local anaesthetics to prevent postherpetic neuralgia: A randomised controlled trial. Lancet.

[9] Dworkin R.H., Turk D.C., Farrar J.T., Haythornthwaite J.A., Jensen M.P., Katz N.P., Kerns R.D., Stucki G., Allen R.R., Bellamy N. (2005). Core outcome measures for chronic pain clinical trials: IMMPACT recommendations. Pain.

[10] Turk D.C., Dworkin R.H., Allen R.R., Bellamy N., Brandenburg N., Carr D.B., Cleeland C., Dionne R., Farrar J.T., Galer B.S. (2003). Core outcome domains for chronic pain clinical trials: IMMPACT recommendations. Pain.

[11] Dworkin R.H., Turk D.C., Wyrwich K.W., Beaton D., Cleeland C.S., Farrar J.T., Haythornthwaite J.A., Jensen M.P., Kerns R.D., Ader D.N. (2008). Interpreting the clinical importance of treatment outcomes in chronic pain clinical trials: IMMPACT recommendations. J. Pain.

[12] Petersen K.L., Rowbotham M.C. (2010). Natural history of sensory function after herpes zoster. Pain.

[13] Dworkin R.H., Gnann J.W., Oaklander A.L., Raja S.N., Schmader K.E., Whitley R.J. (2008). Diagnosis and assessment of pain associated with herpes zoster and postherpetic neuralgia. J. Pain.

[14] Adriaansen E.J.M., Jacobs J.G., Vernooij L.M., van Wijck A.J., Cohen S.P., Huygen F.J.P.M., Rijsdijk M. (2025). 8. Herpes zoster and post herpetic neuralgia. Pain Pract..

[15] Haanpää M., Laippala P., Nurmikko T. (2000). Allodynia and pinprick hypesthesia in acute herpes zoster, and the development of postherpetic neuralgia. J. Pain Symptom Manag..

[16] Kim E.D., Bak H.H., Jo D.H., Park H.J. (2018). Clinical efficacy of transforaminal epidural injection for management of zoster-associated pain: A retrospective analysis. Skelet. Radiol..

[17] Nahm F.S., Choi E., Han W.K., Lee H.J., Gil H.Y., Kim J.H., Lee P.B., Ju H. (2021). Transforaminal epidural steroid injection for zoster-related pain: The golden period for the best outcome. Pain Physician.

[18] Boonstra A.M., Preuper H.R.S., Balk G.A., Stewart R.E. (2014). Cut-off points for mild, moderate, and severe pain on the visual analogue scale for pain in patients with chronic musculoskeletal pain. Pain.

[19] Zelman D.C., Dukes E., Brandenburg N., Bostrom A., Gore M. (2005). Identification of cut-points for mild, moderate and severe pain due to diabetic peripheral neuropathy. Pain.

[20] Moore R.A., Straube S., Aldington D. (2013). Pain measures and cut-offs-‘no worse than mild pain’ as a simple, universal outcome. Anaesthesia.

[21] Choi E.M., Chung M.H., Jun J.H., Chun E.H., Jun I.-J., Park J.H., Choi E.-H., Kim J.E. (2020). Efficacy of intermittent epidural dexamethasone bolus for zoster-associated pain beyond the acute phase. Int. J. Med. Sci..

[22] Park J., Baek S.J., Baek S.H., Kim E.D. (2019). Response to transforaminal epidural block as a useful predictive factor of postherpetic neuralgia. J. Clin. Med..

[23] Luo J., Xiong C. (2013). Youden index and associated cut-points for three ordinal diagnostic groups. Commun. Stat.-Simul. Comput..

[24] Doo A.R., Choi J.-W., Lee J.-H., Kim Y.S., Ki M.-J., Han Y.J., Son J.-S. (2019). The efficacy of selective nerve root block for the long-term outcome of postherpetic neuralgia. Korean J. Pain.

