# Supplementary material for: Time to Epidural Steroid Injection and Complete Remission in Zoster-Associated Pain: A Multicenter Retrospective Cohort Study
Source: Life (Basel). 2026 May 22;16(6):869. doi: 10.3390/life16060869 (PMC13301417; doi:10.3390/life16060869)
Supplement: Supplementary file 1 [file life-16-00869-s001.zip › Supplementary Table S1.pdf]

Supplementary Table S1. Unadjusted Binary and Ordinal Logistic Regression Results for Additional Candidate Variables

Variable	Unadjusted OR (95% CI)	*p*-value
A. Complete remission		
Level (ref: cervical)		
Thoracic	1.172 (0.574–2.394)	0.664
Lumbosacral	0.990 (0.363–2.697)	0.984
Comorbidities		
Hypertension (yes vs. no)	0.702 (0.384–1.285)	0.251
DM (yes vs. no)	0.716 (0.398–1.288)	0.265
Heart disease (yes vs. no)	1.206 (0.415–3.501)	0.731
Thyroid disease (yes vs. no)	1.965 (0.412–9.359)	0.396
CVA (yes vs. no)	0.553 (0.120–2.546)	0.447
Asthma (yes vs. no)	1.277 (0.130–12.531)	0.834
Hyperlipidemia (yes vs. no)	0.207 (0.018–2.321)	0.201
B. Successful response		
Level (ref: cervical)		
Thoracic	1.509 (0.630–3.611)	0.356
Lumbosacral	1.422 (0.394–5.132)	0.591
Comorbidities		
Hypertension (yes vs. no)	0.643 (0.300–1.379)	0.256
DM (yes vs. no)	0.565 (0.265–1.205)	0.140
Heart disease (yes vs. no)	0.926 (0.254–3.380)	0.908
Thyroid disease (yes vs. no)	1.792 (0.221–14.500)	0.585
CVA (yes vs. no)	0.421 (0.078–2.272)	0.315
Asthma (yes vs. no)	1.629 (0.061–43.612)	0.771
Hyperlipidemia (yes vs. no)	1.261 (0.040–39.448)	0.895
C. Ordinal logistic regression		
Level (ref: cervical)		

Thoracic	0.811 (0.404–1.631)	0.557
Lumbosacral	0.941 (0.351–2.519)	0.903
Comorbidities		
Hypertension (yes vs. no)	1.449 (0.801–2.621)	0.220
DM (yes vs. no)	1.457 (0.818–2.596)	0.201
Heart disease (yes vs. no)	0.867 (0.305–2.460)	0.788
Thyroid disease (yes vs. no)	0.514 (0.109–2.417)	0.399
CVA (yes vs. no)	1.970 (0.459–8.457)	0.362
Asthma (yes vs. no)	0.674 (0.064–7.081)	0.743
Hyperlipidemia (yes vs. no)	2.233 (0.255–19.562)	0.468

Values are presented as unadjusted ORs (95% CIs). Panels A and B were analyzed using binary logistic regression, and Panel C using ordinal logistic regression. Variables shown were examined as additional candidate predictors; results for the predefined covariates (time to ESI, age, and sex) are presented in Table 2. These additional candidate variables were examined in exploratory unadjusted analyses and were not included in the primary predefined multivariable models. Baseline VAS score was not evaluated as an additional candidate predictor because baseline pain intensity was incorporated into the binary and ordered outcome definitions through the requirement for ≥50% reduction from baseline. In Panel C, OR >1 indicates higher odds of belonging to a worse outcome tier. Reference category for dermatomal level: cervical.

CI, confidence interval; CVA, cerebrovascular accident; DM, diabetes mellitus; ESI, epidural steroid injection; OR, odds ratio; ref, reference; VAS, visual analog scale.
