# Supplementary material for: Time to Epidural Steroid Injection and Complete Remission in Zoster-Associated Pain: A Multicenter Retrospective Cohort Study
Source: Life (Basel). 2026 May 22;16(6):869. doi: 10.3390/life16060869 (PMC13301417; doi:10.3390/life16060869)
Supplement: Supplementary file 1 [file life-16-00869-s001.zip › Supplementary_STROBE_checklist.pdf]

Supplementary STROBE Checklist

Manuscript: Time to Epidural Steroid Injection and Complete Remission in Zoster-Associated Pain: A Multicenter Retrospective Cohort Study

Authors: Yongsoo Lee, Eun Hee Chun, Hee Yong Kang, Harin Hong, Yeji Yang, Hye Sun Lee and Jung Eun Kim

Study design: cohort study. Page references below correspond to the current manuscript pagination (pp. 1–14).

Item	Section	Checklist item	Page(s)	Location in manuscript
1a	Title/Abstract	Indicate the study design with a commonly used term in the title or abstract.	p.1	Title states ‘A Multicenter Retrospective Cohort Study’; abstract also states ‘multicenter retrospective cohort study’.
1b	Title/Abstract	Provide in the abstract an informative and balanced summary of what was done and what was found.	p.1	Structured summary of background, methods, results, and conclusions.
2	Introduction	Explain the scientific background and rationale for the investigation.	pp.1–2	Introduction paragraphs 1–3.
3	Introduction	State specific objectives, including any prespecified hypotheses.	p.2	Final paragraph of Introduction states aims, primary and secondary endpoints.
4	Methods	Present key elements of study design early in the paper.	p.2	Section 2.1 identifies a retrospective multicenter cohort study.
5	Methods	Describe the setting, locations, and relevant dates, including periods of recruitment/exposure/follow-up/data collection.	p.2	Two university-affiliated hospitals in Seoul; patients treated between March 2018 and February 2025.
6a	Methods	Give eligibility criteria and sources/methods of participant selection; describe follow-up methods.	pp.2–3	Eligibility/exclusion criteria, consecutive screening, required visit windows, and outcome availability are described in Section 2.2.
6b	Methods	For matched studies, give matching criteria and numbers of exposed/unexposed.	N/A	This is not a matched cohort study.
7	Methods	Clearly define outcomes, exposures, predictors, potential confounders, and effect modifiers.	pp.2–4	Time to ESI as exposure; complete remission/successful response/ordered outcome definitions; age and sex as covariates.
8	Methods	For each variable of interest, give data sources and details of assessment; describe comparability of assessment methods.	pp.2–3	Medical-record extraction, standardized bedside sensory assessment (filament brush, alcohol swab, pinprick), and harmonized institutional protocols are described. Independent adjudication by two attending pain physicians with third-adjudicator resolution and high inter-rater agreement (Cohen’s $\kappa = 0.87$) are reported.
9	Methods	Describe efforts to address potential sources of bias.	pp.2–4, 11	Potential bias was addressed through consecutive patient screening, predefined eligibility criteria, harmonized treatment and assessment protocols, independent sensory adjudication, age/sex-adjusted models, and complete-case analysis; residual indication bias is acknowledged in the limitations.
10	Methods	Explain how the study size was arrived at.	pp.4–5	Study size reflected the number of patients screened during the study period who met the eligibility criteria and were included in the final analytic cohort; 332 patients were screened, 250 were eligible, and 215 were included in the analysis (Figure 1).

Item	Section	Checklist item	Page(s)	Location in manuscript
11	Methods	Explain handling of quantitative variables in the analyses, including groupings and rationale when relevant.	pp.2–4	Continuous time-to-ESI modeling, 30-day grouping, 22/42-day exploratory thresholds, and VAS handling are described.
12a	Methods	Describe all statistical methods, including those used to control for confounding.	pp.3–4	Nonparametric tests, LMM, logistic/ordinal regression, covariate adjustment for age/sex, Bonferroni correction, complete-case analysis.
12b	Methods	Describe methods used to examine subgroups and interactions.	pp.3–4	Group × time interaction and interaction contrasts are described; ordered and threshold analyses are also reported.
12c	Methods	Explain how missing data were addressed.	pp.3–5	Complete-case analysis with no imputation; exclusions for missing prespecified outcome data are described in Methods and Figure 1.
12d	Methods	If applicable, explain how loss to follow-up was addressed.	pp.4–5	Complete-case cohort by design; patients with incomplete follow-up data were excluded prior to analysis (Figure 1, p.5).
12e	Methods	Describe any sensitivity analyses.	—	No formal sensitivity analyses were prespecified.
13a	Results	Report numbers of individuals at each stage of the study.	pp.4–5	Screened, excluded, eligible, final analytic cohort, and early/delayed groups are reported.
13b	Results	Give reasons for non-participation/non-inclusion at each stage.	p.5	Reasons for exclusion at screening and from final cohort are shown in Figure 1.
13c	Results	Consider use of a flow diagram.	p.5	Figure 1 presents participant flow.
14a	Results	Give characteristics of study participants and information on exposures/confounders.	pp.5–6, 9–10	Table 1 reports baseline characteristics by conventional 30-day criterion; Table 3 reports characteristics across outcome categories.
14b	Results	Indicate number of participants with missing data for each variable of interest.	p.5	All 215 patients in the final analytic cohort had complete prespecified outcome data by design. Reasons for exclusion due to missing outcome data (n = 13) are detailed in Figure 1.
15	Results	Report numbers of outcome events or summary measures over time.	pp.6–10	12-week complete remission and successful response, longitudinal VAS trajectory, and ordered outcome distribution are reported.
16a	Results	Give unadjusted and, if applicable, adjusted estimates with precision, making clear which confounders were adjusted for.	pp.8–10	Table 2 provides unadjusted and adjusted ORs with 95% CIs; age and sex are stated as adjusted covariates.
16b	Results	Report category boundaries when continuous variables were categorized.	pp.3–4, 7–8	Early/delayed groups (<30 vs ≥30–≤180 days) and exploratory thresholds (<22, 22–<42, ≥42 days) are specified.
16c	Results	When relevant, consider translating relative-risk estimates into absolute risk for a meaningful time period.	p.6	Risk differences and risk ratios are reported for the conventional 30-day comparison at 12 weeks.
17	Results	Report other analyses done, such as subgroup analyses, interactions, and sensitivity analyses.	pp.6–10	Section 3.4 reports interaction contrasts from the linear mixed model; Sections 3.5 and 3.6 report exploratory threshold analysis, ordinal regression, and outcome-group comparisons. No sensitivity analysis is explicitly reported in the current manuscript.
18	Discussion	Summarize key results with reference to study objectives.	pp.10–11	Opening Discussion paragraphs directly interpret results against the stated aims.

Item	Section	Checklist item	Page(s)	Location in manuscript
19	Discussion	Discuss limitations of the study, considering potential bias or imprecision.	p.12	The limitations paragraph discusses the retrospective design, indication bias, nonstandardized concomitant pharmacologic management, and limited 12-week follow-up.
20	Discussion	Give a cautious overall interpretation considering objectives, limitations, multiplicity of analyses, and relevant evidence.	pp.10–13	Interpretation is linked to prior literature, exploratory thresholds, pathophysiology, strengths, and limitations.
21	Discussion	Discuss the generalisability of the study results.	pp.10–13	Multicenter university-hospital context is clear, but external validity is not discussed explicitly in a dedicated statement.
22	Other information	Give the source of funding and the role of the funders.	p.13	Funding source and funder noninvolvement are stated in the end matter.
